# Diagnostic efficacy of long non-coding RNAs in multiple sclerosis: a systematic review and meta-analysis

**DOI:** 10.3389/fgene.2024.1400387

**Published:** 2024-05-15

**Authors:** Yongdong Wang, Jing Wang, Xinyin Zhang, Chengyan Xia, Zhiping Wang

**Affiliations:** Department of Neurology, Chengdu Shuangliu District Hospital of Traditional Chinese Medicine, Chengdu, Sichuan, China

**Keywords:** long non-coding RNA, multiple sclerosis, systematic review, meta analysis, diagnosis

## Abstract

**Background:**

Currently, an increasing body of research suggests that blood-based long non-coding RNAs (lncRNAs) could serve as biomarkers for diagnosing multiple sclerosis (MS). This meta-analysis evaluates the diagnostic capabilities of selected lncRNAs in distinguishing individuals with MS from healthy controls and in differentiating between the relapsing and remitting phases of the disease.

**Methods:**

We conducted comprehensive searches across seven databases in both Chinese and English to identify relevant studies, applying stringent inclusion and exclusion criteria. The quality of the selected references was rigorously assessed using the QUADAS-2 tool. The analysis involved calculating summarized sensitivity (SSEN), specificity (SSPE), positive likelihood ratio (SPLR), negative likelihood ratio (SNLR), and diagnostic odds ratio (DOR) with 95% confidence intervals (CIs). Accuracy was assessed using summary receiver operating characteristic (SROC) curves.

**Results:**

Thirteen high-quality studies were selected for inclusion in the meta-analysis. Our meta-analysis assessed the combined diagnostic performance of lncRNAs in distinguishing MS patients from healthy controls. We found a SSEN of 0.81 (95% CI: 0.74–0.87), SSPE of 0.84 (95% CI: 0.78–0.89), SPLR of 5.14 (95% CI: 3.63–7.28), SNLR of 0.22 (95% CI: 0.16–0.31), and DOR of 23.17 (95% CI: 14.07–38.17), with an AUC of 0.90 (95% CI: 0.87–0.92). For differentiating between relapsing and remitting MS, the results showed a SSEN of 0.79 (95% CI: 0.71–0.85), SSPE of 0.76 (95% CI: 0.64–0.85), SPLR of 3.34 (95% CI: 2.09–5.33), SNLR of 0.28 (95% CI: 0.19–0.40), and DOR of 12.09 (95% CI: 5.70–25.68), with an AUC of 0.84 (95% CI: 0.81–0.87).

**Conclusion:**

This analysis underscores the significant role of lncRNAs as biomarkers in MS diagnosis and differentiation between its relapsing and remitting forms.

## Introduction

Multiple sclerosis (MS) is a chronic autoimmune inflammatory demyelinating disorder that targets the central nervous system. Its incidence exhibits substantial geographical and ethnic variability. In Caucasian populations across Europe, North America, and Oceania, the incidence rate is comparatively elevated, surpassing 100 per 100,000 individuals (Bartulos Iglesias et al., 2015; Howard et al., 2016; Dal et al., 2023). Conversely, the incidence in Asian populations is markedly lower. A comprehensive, nationwide hospital-based study in China reported an annual MS incidence of 0.235 per 100,000, with 0.055 per 100,000 in children and 0.288 per 100,000 in adults, underscoring a significantly reduced incidence in comparison to Caucasian groups (Tian et al., 2020). Importantly, recent evidence suggests a notable uptrend in MS incidence (Koch-Henriksen and Magyari, 2021). The prognosis of MS is highly individualized, contingent on disease type, symptom severity, and treatment responsiveness. Clinical trajectories can range from gradual symptom progression to rapid deterioration. Timely diagnosis and intervention are imperative for mitigating disease advancement, managing symptoms, and enhancing life quality.

The diagnostic process for MS generally adheres to the established “McDonald Criteria” (Thompson et al., 2018). These criteria necessitate evidence of lesions dispersed across the central nervous system (termed “dissemination”) and manifesting over time (“temporal”). Principal diagnostic tools encompass Magnetic Resonance Imaging for detecting brain and spinal cord lesions, cerebrospinal fluid analysis for immune system activity indicators, and clinical symptom evaluation. MS diagnosis also involves the exclusion of other potential conditions. Given the complexity of MS diagnosis, blood markers have emerged as a non-invasive and efficient alternative, significantly aiding in MS diagnosis. For instance, TLR-2 and TLR-4 expression in peripheral blood demonstrate high sensitivity and specificity in differentiating MS patients from healthy individuals (Labib et al., 2022). A meta-analysis encompassing 9 studies indicated that serum miRNA may serve as a viable diagnostic biomarker for MS (Zailaie et al., 2022).

Non-coding RNA, transcribed from the genome but not involved in protein coding, plays a pivotal role in transcriptional and post-transcriptional regulation and serves as a crucial epigenetic modulator (Nowak et al., 2022). Contemporary research predominantly focuses on regulatory non-coding RNAs, categorized based on length into small non-coding RNAs (sncRNAs), medium-length non-coding RNAs (mncRNAs), and long non-coding RNAs (lncRNAs) (Dahariya et al., 2019). The lncRNA present in blood has been established as a non-invasive biomarker, demonstrating significant potential in the diagnosis of a diverse array of diseases (Li et al., 2022; Liao et al., 2022; Zhao et al., 2022). Recent findings suggest that lncRNAs could serve as potential biomarkers in MS, predicting disease onset, activity, progression stage, and response to disease-modifying drugs (Nociti and Santoro, 2021). For example, a study involving 30 healthy controls and 120 MS patients identified serum lncRNA RUNXOR as a discriminative marker between clinically isolated syndrome and relapsing-remitting multiple sclerosis (Haridy et al., 2023). Another study highlighted the diagnostic potential of AL928742.12 and RP11-530C5.1 as MS biomarkers (Ghoveud et al., 2020). This meta-analysis aims to verify the consistency of lncRNA in diagnosing MS and to possibly uncover trends or patterns not discernible in individual studies.

## Materials and methods

### Literature search

Comprehensive literature searches were executed in English databases, including PubMed, EMBASE, the Cochrane Library, and Web of Science, alongside Chinese databases such as China National Knowledge Infrastructure, Wanfang Data Knowledge Service Platform, and the Chongqing VIP Chinese Scientific Journals Database. These searches focused on lncRNA expression and MS-related research. A strategic combination of Medical Subject Headings (MESH) was employed to ensure an exhaustive coverage of relevant literature. The temporal scope of the search spanned from the inception of each database up to 1 December 2023, encompassing literature in both English and Chinese. Detailed Boolean logic retrieval formulas utilized in each database are presented as follows:

(Noncoding RNA, Long) OR (lncRNA)) OR (Long ncRNA)) OR (ncRNA, Long)) OR (RNA, Long Non-Translated)) OR (Long Non-Translated RNA)) OR (Non-Translated RNA, Long)) OR (RNA, Long Non Translated)) OR (Long Non-Coding RNA)) OR (Long Non Coding RNA)) OR (Non-Coding RNA, Long)) OR (RNA, Long Non-Coding)) OR (Long Non-Protein-Coding RNA)) OR (Long Non Protein Coding RNA)) OR (Non-Protein-Coding RNA, Long)) OR (RNA, Long Non-Protein-Coding)) OR (Long Noncoding RNA)) OR (RNA, Long Untranslated)) OR (Long Untranslated RNA)) OR (Untranslated RNA, Long)) OR (Long ncRNAs)) OR (ncRNAs, Long)) OR (Long Intergenic Non-Protein Coding RNA)) OR (Long Intergenic Non Protein Coding RNA)) OR (LincRNAs)) OR (LINC RNA)) OR (LincRNA)) AND (Sclerosis, Multiple) OR (Sclerosis, Disseminated)) OR (Disseminated Sclerosis)) OR (MS (Multiple Sclerosis))) OR (Multiple Sclerosis, Acute Fulminating)) OR (Multiple sclerosis)).

### Inclusion and exclusion criteria

Inclusion Criteria: 1. Studies examining lncRNAs in blood (including plasma and serum) related to the diagnosis or subtyping of MS, excluding other bodily fluids or tissues. 2. Inclusion of patients with definitive MS diagnosis, excluding clinically suspected cases. 3. Availability or calculability of diagnostic test data: true positive (TP), false positive (FP), true negative (TN), and false negative (FN). 4. Presence of a control group for comparison, either healthy individuals or contrasting MS relapse and remission phases. 5. A minimum sample size of 20 patients. 6. Publications in English or Chinese.

Exclusion Criteria: 1. Studies lacking derivable diagnostic test data, including inability to obtain such data via direct or indirect means. 2. Non-original data literature, such as reviews, commentaries, and conference abstracts. 3. Research based on animal or cell models. 4. Studies focusing on children or infants, rather than adult populations.

### Data extraction and quality assessment

Two independent investigators conducted the literature search and data extraction, with any disagreements resolved through discussion with a third author. Extracted data primarily included the first author’s name, year of publication, study country, language, basic demographics (age, gender) of MS patients and controls, sample source, lncRNA species, internal reference, cut-off values, and diagnostic test data (TP, FP, TN, FN). The Quality Assessment of Diagnostic Accuracy Studies-2 (QUADAS-2) tool was employed for evaluating the quality of included studies, focusing on four critical domains: patient selection, index test, reference standard, and flow and timing (Whiting et al., 2011).

### Statistical analysis

Diagnostic values from the included literature were meta-analyzed using Stata 15.0 and Meta-DiSc 1.4 (Zamora et al., 2006), while Review Manager 5.4 facilitated the literature quality assessment. Spearman regression analysis was applied to identify any threshold effect. In the absence of a threshold effect, Cochran’s Q test and Higgins I-squared test were employed for heterogeneity assessment. A bivariate random-effects model (Negeri and Beyene, 2020; Baragilly and Willis, 2022) calculated the summarized sensitivity (SSEN), specificity (SSPE), positive likelihood ratio (SPLR), negative likelihood ratio (SNLR), and diagnostic odds ratio (DOR). Summary receiver operating characteristic (SROC) curves were generated, and the area under the curve (AUC) was computed for diagnostic accuracy assessment. Fagan’s nomogram (Caraguel and Vanderstichel, 2013) was utilized for post-test probability verification, while sensitivity analysis gauged the robustness of results against individual studies. The Deek’s test ascertained the presence of publication bias. Meta-regression and subgroup analyses were conducted to explore potential heterogeneity sources. A p-value of less than 0.05 was considered statistically significant.

## Results

### Literature search and quality assessment

**FIGURE 1:**
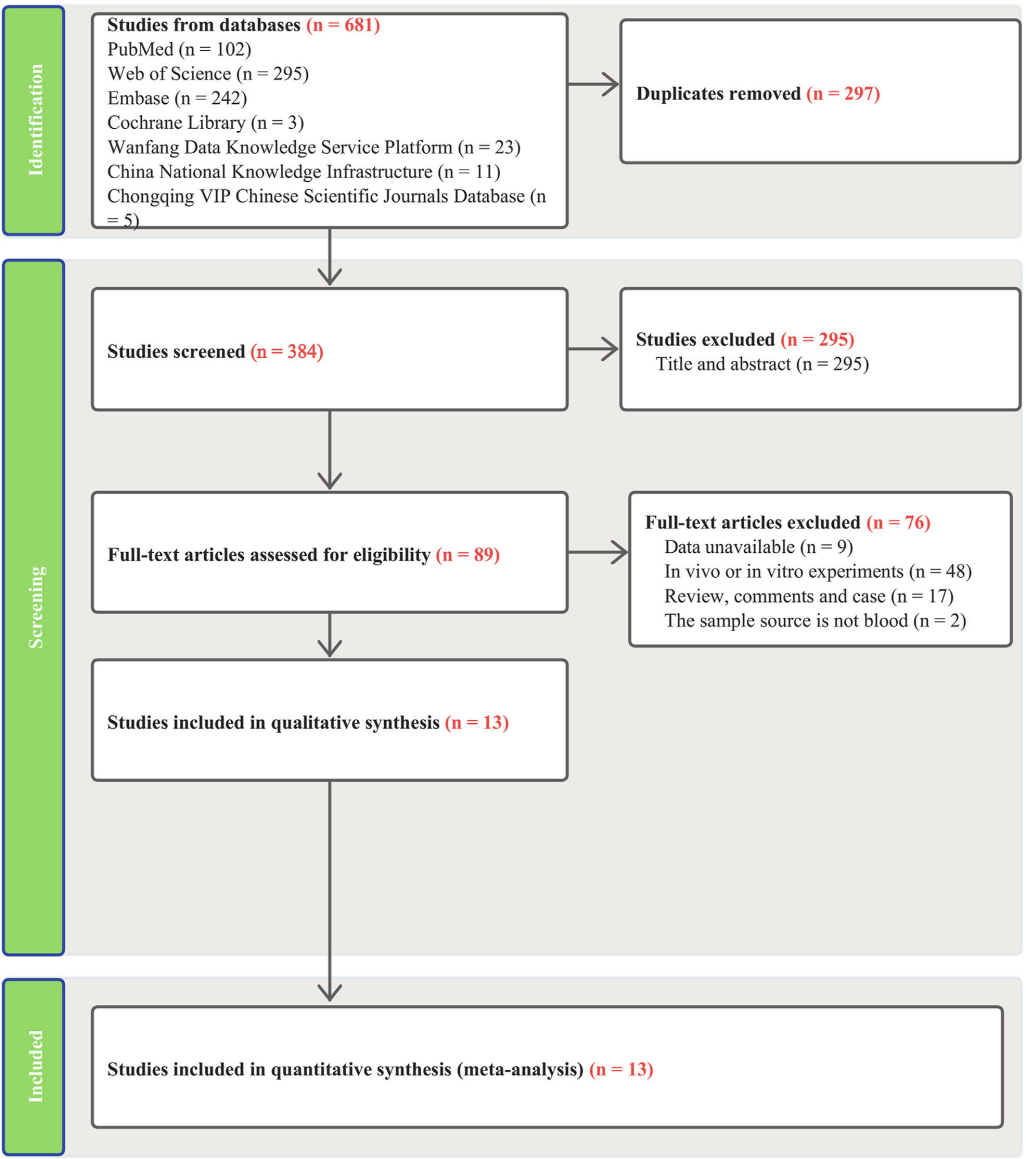
Flow chart for inclusion and exclusion of literature.

**FIGURE 2:**
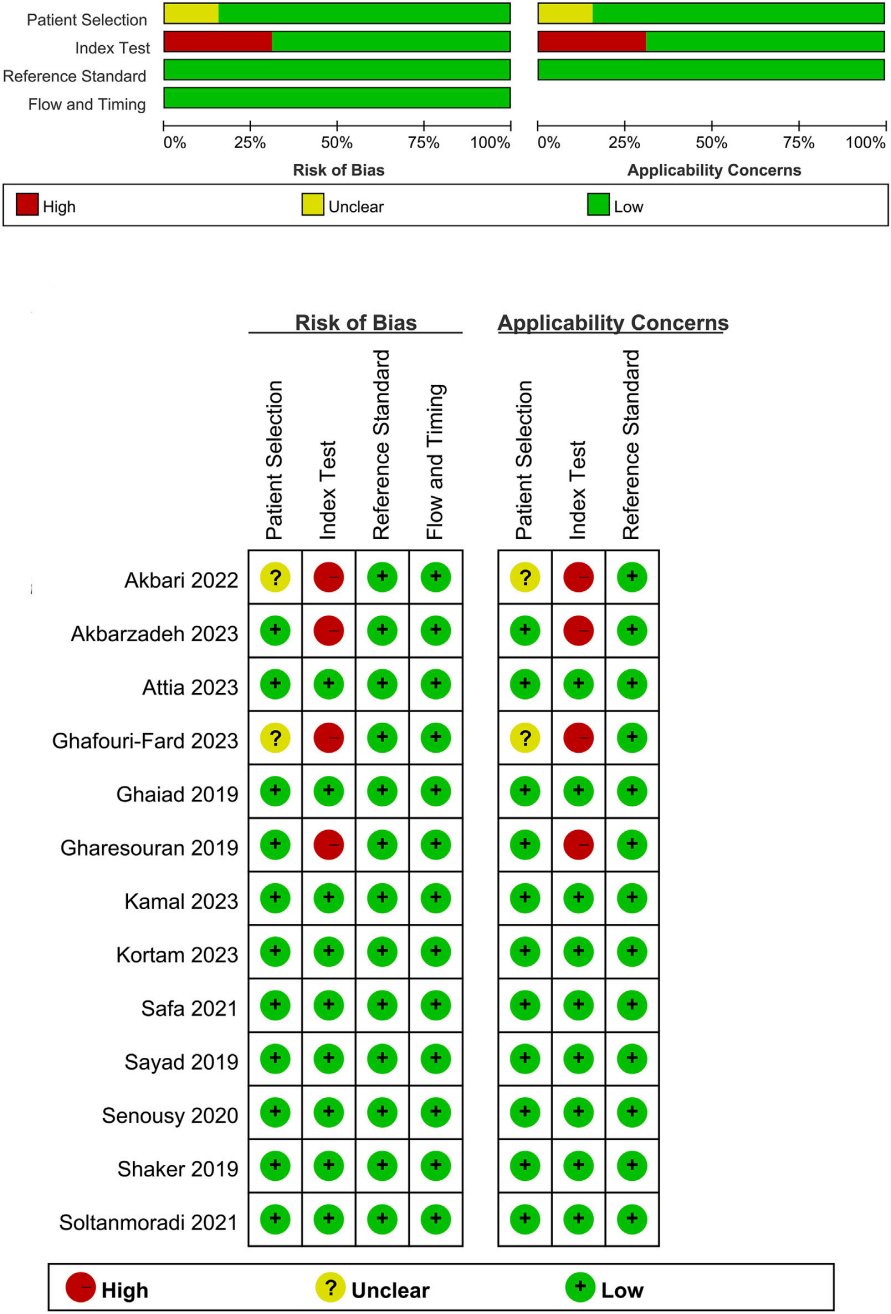
Quality assessment of included studies using QUADAS-2. This figure presents the quality assessment of each included study using the QUADAS-2 tool, which evaluates the risk of bias and applicability concerns across four domains: patient selection, index test, reference standard, and flow and timing. Each study is assessed for potential bias and applicability with the following indicators: green with a plus sign (+) denotes low risk of bias or concern, yellow with a question mark (?) indicates unclear risk or concern, and red with a minus sign (−) represents high risk or significant concern.

Our search across four English and three Chinese databases yielded 681 articles. After duplicates were removed, 384 articles remained. Subsequent careful screening of abstracts led to the exclusion of 295 articles. Final inclusion criteria—excluding animal cell experiments, non-blood sample studies, data extraction issues, reviews, and comments—resulted in 13 studies (Gharesouran et al., 2019b; Sayad et al., 2019; Shaker et al., 2019; Ghaiad et al., 2020; Senousy et al., 2020; Safa et al., 2021; Soltanmoradi et al., 2021; Akbari et al., 2022; Amiri et al., 2022; Attia et al., 2023; Ghafouri-Fard et al., 2023; Kamal et al., 2023; Kortam et al., 2023) qualifying for the meta-analysis. This process is detailed in Figure 1. The studies, published from 2019 to 2023, predominantly originate from Egypt and Iran and are written in English. The main details of these studies are summarized in Table 1. Quality assessment via QUADAS-2 revealed some risks in patient selection due to missing age and gender details in control groups and high risk in the index test due to absent specific cutoff values. Nevertheless, the overall literature quality is high (Figure 2).

**TABLE 1:** The basic characteristics of the included studies.

First author	Year	Country	No. of MS patients	Age	Sex (Male/Female)	No. of control	Age	Sex (Male/Female)	Sample type	LncRNA	Test method	Normalizer
Ghaiad et al.	2019	Egypt	72	Relapse: 33.8 (14.3)	Relapse: 13/24	28	39 (6)	9/19	Blood	APOA1-AS, IFNG-AS1, RMRP	qRT-PCR	GAPDH
				Remission: 35.5 (13.3)	Remission: 8/27							
Gharesouran et al.	2019	Iran	50	36.2 ± 2.9	19/31	50	35.3 ± 2.1	23/27	Blood	HUR1	qRT-PCR	GAS5
Sayad et al.	2019	Iran	50	36.2 ± 2.7	15/35	50	35.3 ± 2.4	15/35	Blood	HULC	qRT-PCR	HPRT1
Shaker et al.	2019	Egypt	45	31.3 ± 8.3	6/39	45	32.4 ± 9.2	8/37	Blood	MALAT1, lnc-DC	qRT-PCR	GAPDH
Senousy et al.	2020	Egypt	108	31.23 ± 8.57	24/84	104	32.9 ± 9.66	26/78	Serum	GAS5	qRT-PCR	GAPDH
Safa et al.	2021	Iran	50	65.30 ± 10.80	12/38	50	6 4. 2 0 ± 1 0. 5 0	13/37	Blood	HNF1A-AS1, LINC00305, LNC-MKI67IP	qRT-PCR	Beta-2-microglobulin
Soltanmoradi et al.	2021	Iran	60	Relapse: 30 ± 7.5	Relapse: 5/25	30	29 ± 5.2	6/24	Blood	NKILA, H19, HOTAIR, THRIL, ANRIL	qRT-PCR	Beta-2-microglobulin
				Remission: 29 ± 6.3	Remission: 6/24							
Akbari et al.	2022	Iran	50	Male: 37.5 ± 10.8	12/38	50	NA	NA	Blood	SNHG5, DANCR	qRT-PCR	Beta-2-microglobulin
				Female: 40.13 ± 9.52								
Akbarzadeh et al.	2023	Iran	95	36.81 ± 8.37	15/80	95	36.81 ± 8.35	15/80	Blood	MIAT, H19, NRON	qRT-PCR	GAPDH
Attia et al.	2023	Egypt	70	Relapse: 35 (20–50)	Relapse: 9/26	30	30 (22–45)	10/20	Serum	lnc-EGFR	qRT-PCR	GAPDH
				Remission: 32 (19–48)	Remission: 11/24							
Ghafouri-Fard et al.	2023	Iran	50	Male: 37.5 ± 10.8	12/38	50	NA	NA	Blood	RAD51-AS1, ZNRD1ASP, NORAD	qRT-PCR	GAPDH
				Female: 40.13 ± 9.52								
Kamal et al.	2023	Egypt	118	31.10 ± 9.04 et al.	57/61	20	37.70 ± 16.84	9/11	Blood	MAGI2-AS3	qRT-PCR	GAPDH
Kortam et al.	2023	Egypt	100	32 ± 8.9	22/78	50	35 ± 9.7	16/34	Blood	MAGI2-AS3	qRT-PCR	GAPDH

### Efficacy of blood lncRNAs in differentiating MS patients

#### MS patients vs. healthy individuals

**FIGURE 3:**
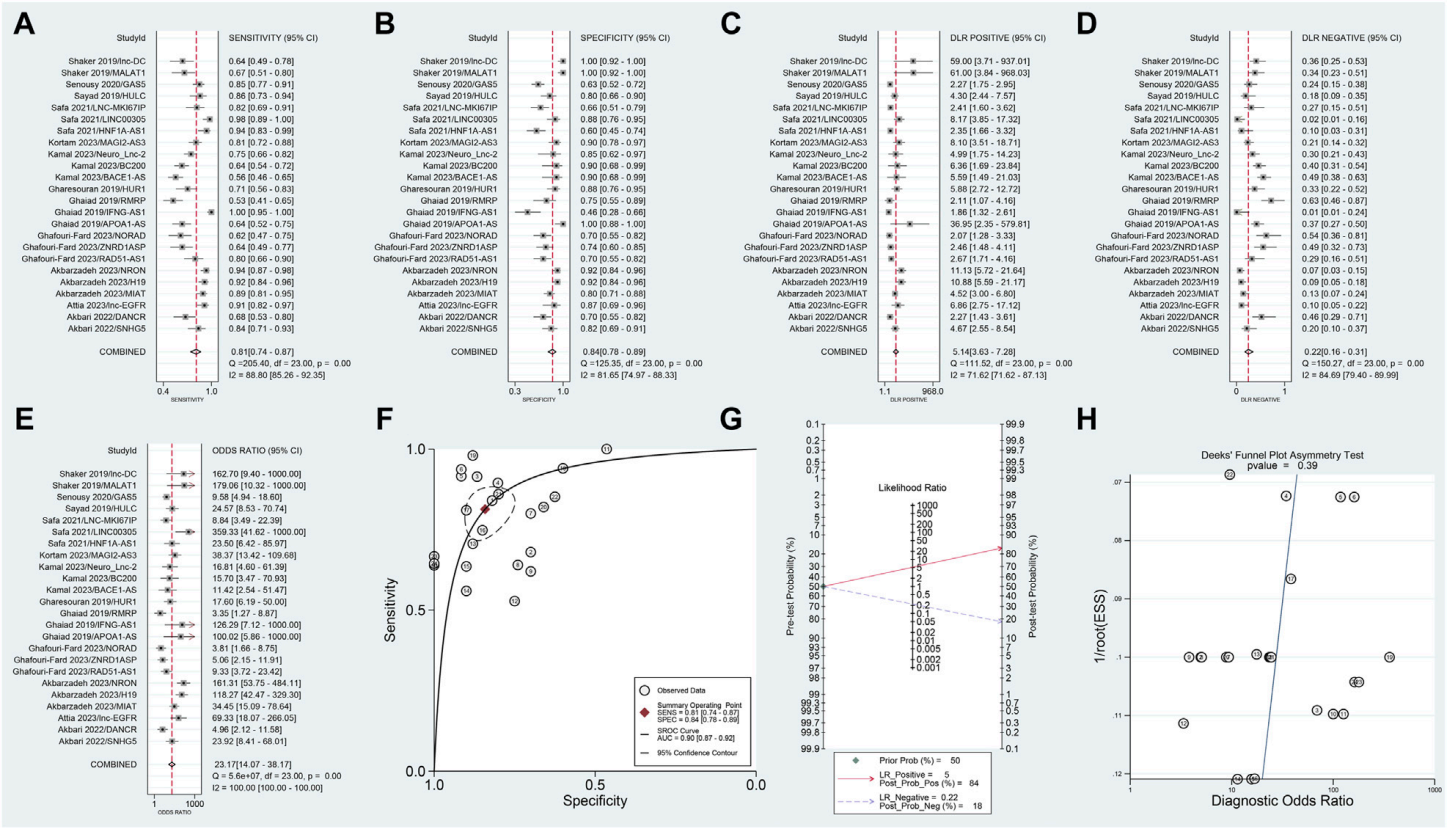
Diagnostic evaluation of blood lncRNAs in MS using meta-Analytic techniques. This figure illustrates the meta-analytic assessment of blood lncRNAs in differentiating MS patients from healthy controls. Forest plots of **(A)** SSEN, **(B)** SSPE, **(C)** SPLR, **(D)** SNLR and **(E)** DOR. **(F)** AUC of SROC. **(G)** The fagan’s nomogram. **(H)** The results of publication bias.

In this meta-analysis, which included data from 12 articles and 24 studies, we observed no threshold effect (Spearman correlation coefficient: 0.230, *p* = 0.280), indicating consistency among the studies. The pooled SSEN was 0.81 (95% CI 0.74–0.87) (Figure 3A) and SSPE was 0.84 (95% CI 0.78–0.89) (Figure 3B), suggesting high accuracy of lncRNA testing in identifying MS. The SPLR of 5.14 (95% CI 3.63–7.28) (Figure 3C) implies that MS patients are significantly more likely to test positive compared to healthy individuals, while the SNLR of 0.22 (95% CI 0.16–0.31) (Figure 3D) indicates that a negative test is informative for ruling out the disease. The DOR of 23.17 (95% CI 14.07–38.17) (Figure 3E) and the AUC of 0.90 (95% CI 0.87–0.92) (Figure 3F) underline the strong discriminative power of blood lncRNAs. Fagan’s nomogram and the absence of publication bias (Figures 3G, H) further validate the clinical utility of these biomarkers, suggesting their potential integration into diagnostic workflows to improve early detection and management of MS.

#### Relapsing vs. remitting MS patients

**FIGURE 4:**
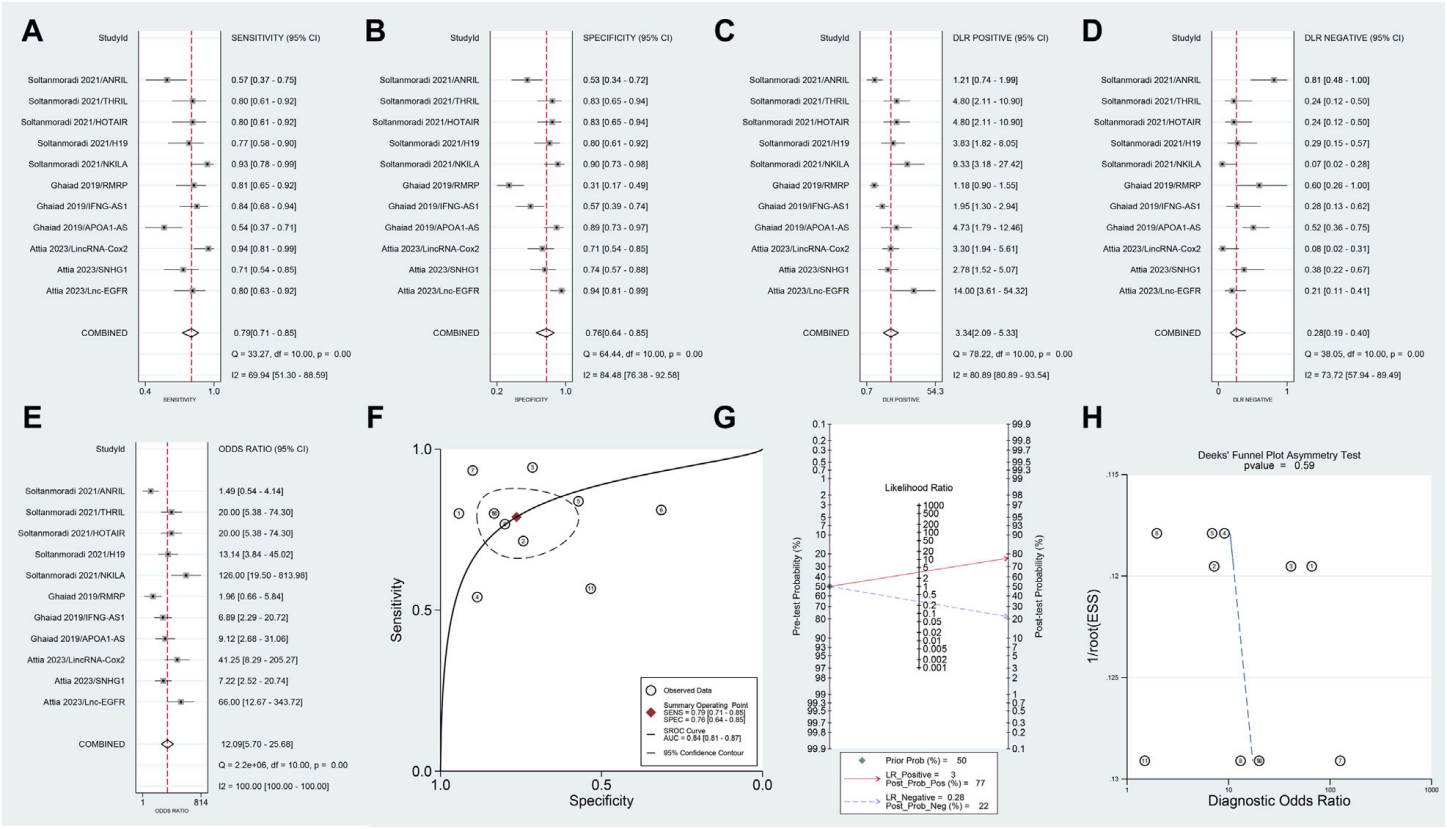
Comparative diagnostic performance of blood lncRNAs in different MS phases. Forest plots in this figure evaluate the effectiveness of blood lncRNAs in distinguishing between relapsing MS and remitting MS patients. Forest plot of **(A)** SSEN, **(B)** SSPE, **(C)** SPLR, **(D)** SNLR and **(E)** DOR. **(F)** AUC of SROC. **(G)** The fagan’s nomogram. **(H)** The results of publication bias.

This segment of the meta-analysis involved three articles comprising 11 studies. The Spearman correlation coefficient was 0.083 (*p* = 0.809), showing no threshold effect, which suggests consistency across the included studies. The meta-analysis yielded a SSEN of 0.79 (95% CI 0.71–0.85) (Figure 4A) and SSPE of 0.76 (95% CI 0.64–0.85) (Figure 4B), indicating high accuracy of lncRNA testing in differentiating conditions. The SPLR was 3.34 (95% CI 2,09–5.33) (Figure 4C), suggesting that positive test results are more likely in affected individuals, while the SNLR of 0.28 (95% CI 0.19–0.40) (Figure 4D) reinforces the reliability of a negative test. The DOR stood at 12.09 (95% CI 5.70–25.68) (Figure 4E), highlighting the strong diagnostic capability of the test. Additionally, the AUC of 0.84 (95% CI 0.81–0.87) (Figure 4F) underscores the overall effectiveness of the lncRNAs as diagnostic markers. Fagan’s nomogram, depicted in Figure 4G, confirmed significant post-test probability changes, enhancing clinical decision-making based on lncRNA testing results. The absence of publication bias, as shown in Figure 4H, further validates the robustness of these findings.

### Meta-regression and subgroup analyses

**FIGURE 5:**
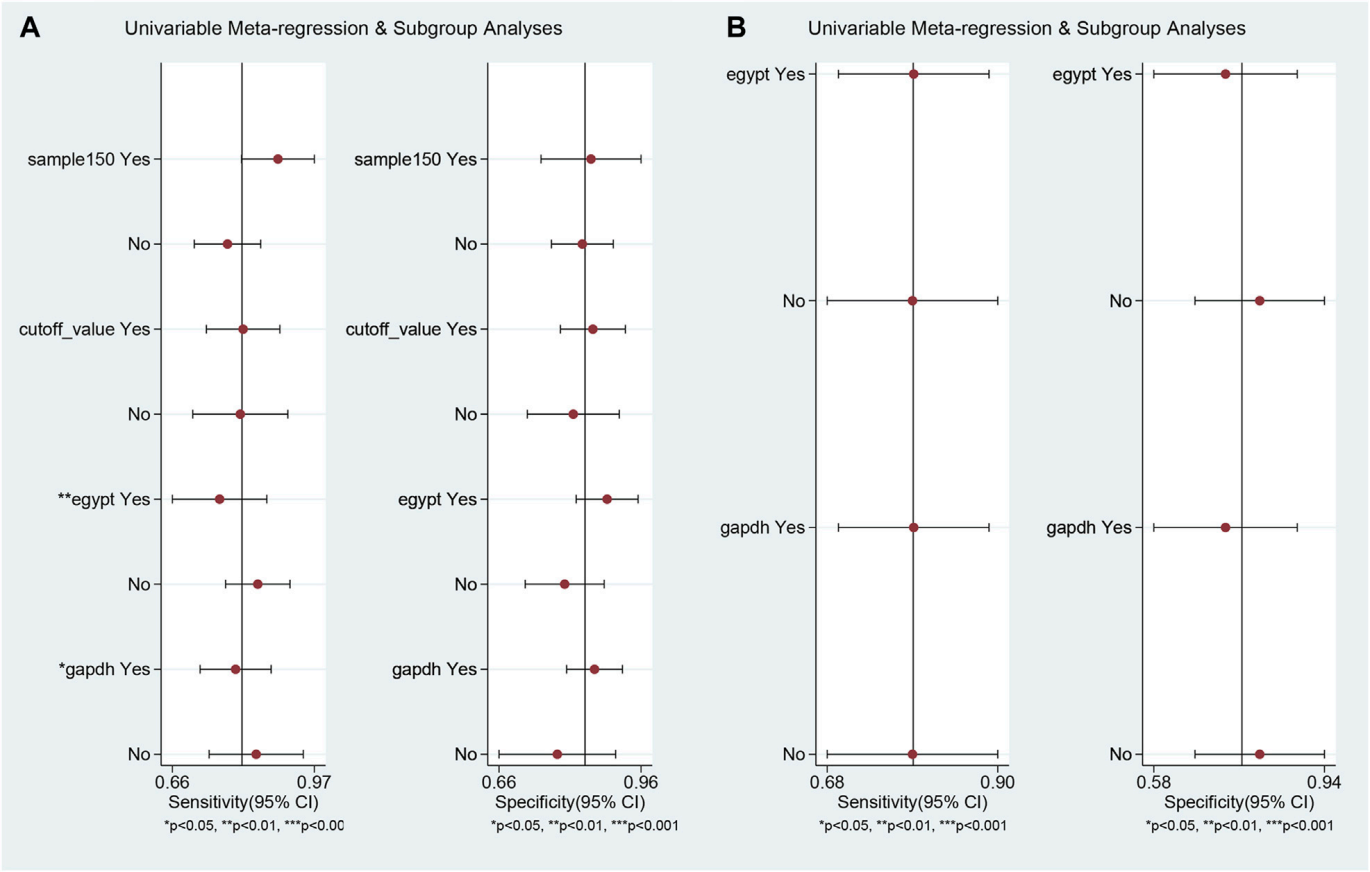
Univariable meta-regression and subgroup analysis results. This figure presents the outcomes of univariable meta-regression and subgroup analyses, assessing the impact of various covariates on the diagnostic performance of blood lncRNAs. **(A)** The blood lncRNAs was utilized to differentiate between MS patients and the healthy control. **(B)** The blood lncRNAs was utilized to differentiate between Relapsing MS and the Remitting MS patients.

Univariable meta-regression and subgroup analyses (Figure 5; Table 2) were conducted. For differentiating MS patients from healthy individuals, we analyzed sample size, cutoff clarity, study origin, and GAPDH usage. Study origin (Egypt) and GAPDH usage emerged as heterogeneity contributors. In differentiating relapsing from remitting patients, neither of these factors significantly impacted heterogeneity.

**TABLE 2:** Meta regression and subgroup analysis of the meta-analysis.

	Parameter	Category	No. of studies	Sensitivity [95% CI]	*p*-Value	Specificity [95% CI]	*p*-Value
MS vs. Health	Sample>150	Yes	5	0.89 [0.81–0.97]	0.48	0.85 [0.75–0.96]	0.15
		No	19	0.78 [0.71–0.85]		0.84 [0.77–0.90]	
	Cut-off value	Yes	15	0.82 [0.74–0.89]	0.06	0.86 [0.79–0.93]	0.12
		No	9	0.81 [0.71–0.91]		0.82 [0.72–0.92]	
	Egypt	Yes	11	0.77 [0.66–0.87]	<0.01	0.89 [0.82–0.96]	0.28
		No	13	0.85 [0.78–0.92]		0.80 [0.71–0.88]	
	GAPDH	Yes	17	0.80 [0.72–0.88]	0.02	0.86 [0.80–0.92]	0.33
		No	7	0.84 [0.74–0.94]		0.78 [0.66–0.91]	
Relapse vs. Remission	Egypt	Yes	6	0.79 [0.69–0.89]	0.10	0.73 [0.58–0.88]	0.17
		No	5	0.79 [0.68–0.90]		0.80 [0.66–0.94]	
	GAPDH	Yes	6	0.79 [0.69–0.89]	0.10	0.73 [0.58–0.88]	0.17
		No	5	0.79 [0.68–0.90]		0.80 [0.66–0.94]	

### Sensitivity analysis

#### MS patients vs. healthy individuals

**FIGURE 6:**
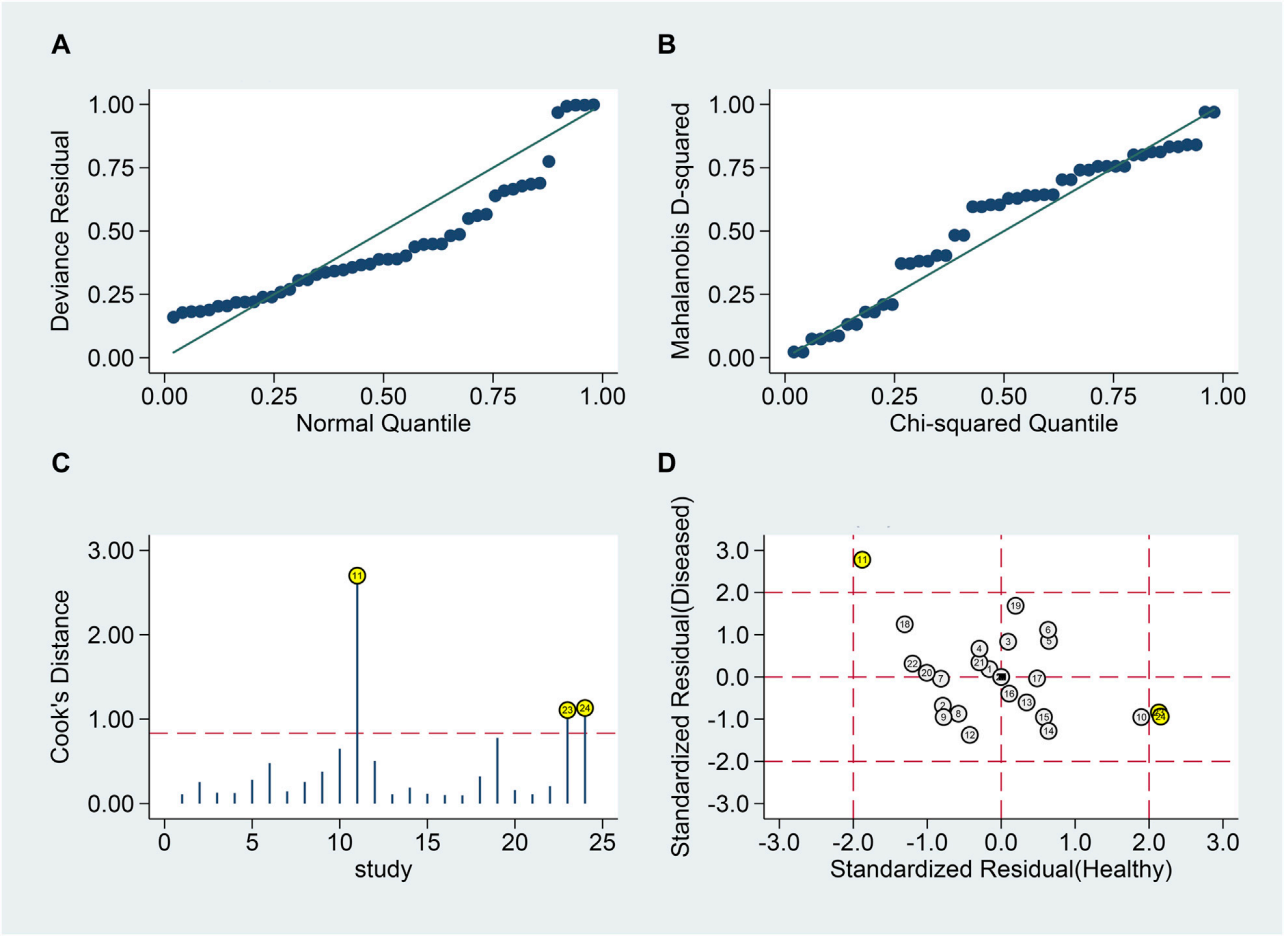
Sensitivity analysis of blood lncRNAs for MS diagnosis. Here, sensitivity analyses are conducted to evaluate the robustness of using blood lncRNAs to distinguish MS patients from healthy controls. **(A)** goodness-of-fit, **(B)** bivariate normality, **(C)** influence analysis, and **(D)** outlier detection.

Goodness of fit and bivariate normality analyses (Figures 6A, B) confirmed model robustness. Influence analysis identified three outliers (Figures 6C, D). Excluding these outliers resulted in SSEN of 0.81 (95% CI 0.74–0.86), SSPE of 0.82 (95% CI 0.76–0.86), SPLR of 4.4 (95% CI 3.3–5.8), SNLR of 0.24 (95% CI 0.17–0.33), and DOR of 18 (95% CI 11–31), with an AUC of 0.88 (95% CI 0.85–0.91).

#### Relapsing vs. remitting MS patients

**FIGURE 7:**
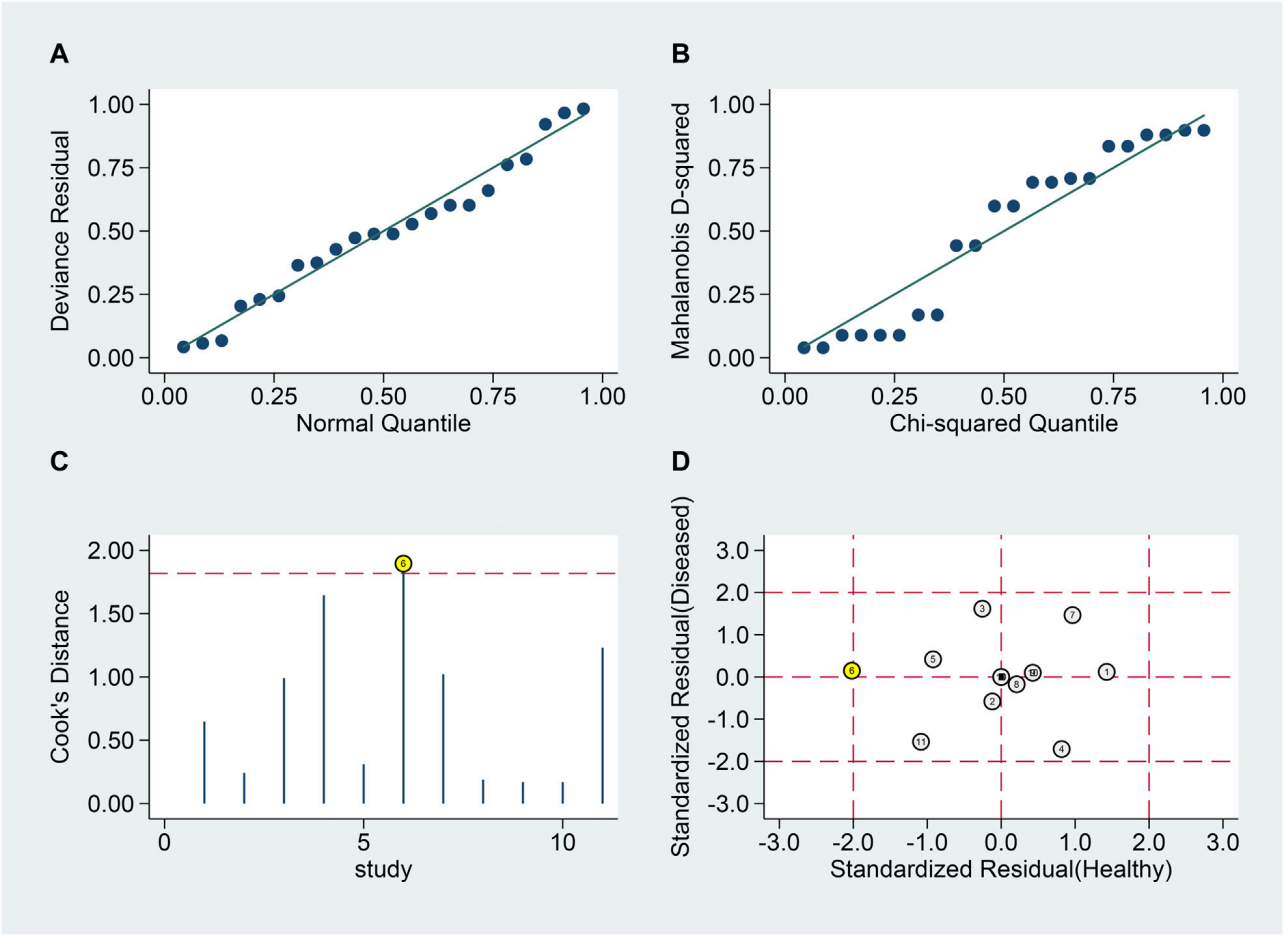
Sensitivity analysis for blood lncRNAs in differentiating MS Phases. Sensitivity analyses are shown assessing the reliability of blood lncRNAs in distinguishing between relapsing and remitting MS patients. **(A)** goodness-of-fit, **(B)** bivariate normality, **(C)** influence analysis, and **(D)** outlier detection.

Similar analyses (Figures 7A, B) identified one outlier (Figures 7C, D). Post-exclusion results showed SSEN of 0.79 (95% CI 0.70–0.86), SSPE of 0.79 (95% CI 0.70–0.86), SPLR of 3.8 (95% CI 2.5–5.8), SNLR of 0.27 (95% CI 0.18–0.40), and DOR of 14 (95% CI 7–30), with an AUC of 0.86 (95% CI 0.83–0.89).

## Discussion

Due to the complexity of clinical characteristics and unclear pathogenesis of MS, there is an urgent need to search for more potential biomarkers for specific diagnosis and prediction of disease progression in patients at this stage. In the field of molecular biology, lncRNAs have received widespread attention. These RNAs can process mRNA encoding proteins and participate in various pathological and physiological processes of organisms, such as immune response, cell proliferation, apoptosis, and autophagy (Wang et al., 2014). Previous studies have shown that lncRNA is closely related to neurological diseases such as Alzheimer’s disease, epilepsy, and MS (Hauser et al., 2018; Chen et al., 2019). Additionally, studies have found significant expression differences of MALAT1 in the peripheral blood of MS patients compared to healthy controls, and MALAT1 demonstrates high diagnostic capability for MS patients (Gharesouran et al., 2019a). To further explore the diagnostic capabilities of lncRNAs, we conducted this meta-analysis.

Our meta-analysis underscores the diagnostic utility of specific lncRNAs in distinguishing relapsing-remitting multiple sclerosis (RRMS) from healthy controls. Biomarkers such as H19, APOA1-AS, IFNG-AS1, RMRP, and Lnc-EGFR exhibit considerable sensitivity and specificity, affirming their potential for clinical applications. To further elucidate their utility, it is imperative to explore these lncRNAs across various MS subtypes and other neuroinflammatory conditions. Such investigations are crucial for verifying whether these biomarkers are exclusive to RRMS or indicative of a broader spectrum of MS pathology. Emerging research has revealed the prognostic capabilities of RUNXOR lncRNA in predicting the progression from clinically isolated syndrome to RRMS, and its potential as a marker for advancing from RRMS to secondary progressive MS (Haridy et al., 2023). Additionally, another study has identified lncRNA biomarkers capable of differentiating phenotypic severity in MS (Gupta et al., 2019). These findings suggest that lncRNAs could play a critical role not only in diagnosing MS but also in forecasting its progression and assessing disease severity. Thus, the integration of these lncRNAs into clinical practice could significantly enhance the management and stratification of MS patients, providing a foundation for personalized therapeutic strategies.

The lncRNAs discovered in this study not only play a diagnostic role but are also related to the pathological mechanisms of MS. For instance, it has been observed that the lncRNA GAS5 is significantly upregulated in the microglia of MS patients (Sun et al., 2017). GAS5 inhibits the polarization of M2 microglia while promoting the polarization of the M1 subtype. The predominance of M1 and the scarcity of M2 microglia are key pathological features of MS (Sun et al., 2017). M1-polarized microglia contribute to neuronal apoptosis and inhibit the differentiation of oligodendrocyte progenitors into mature oligodendrocytes. In contrast, M2-polarized microglia support neuronal survival, dendritic growth, and the differentiation of oligodendrocyte progenitors, suggesting their role in remyelination and neural repair at MS lesions (Miron et al., 2013; Franco and Fernández-Suárez, 2015). In the experimental autoimmune encephalomyelitis (EAE) animal model, significant suppression of inflammatory cytokines from microglia alleviates the severity of EAE, while transplantation of M2-polarized microglia into the central nervous system promotes recovery (Yu et al., 2015). These experimental findings underscore the critical involvement of different microglial polarization states in the pathogenesis of MS. The differential expression of GAS5 in these polarization states suggests that targeting microglial polarization might be a novel therapeutic approach for MS, centered on microglial dynamics.

MALAT1, located in the cell nucleus, orchestrates the expression of various genes by arranging ribonucleoprotein complexes, thereby influencing RNA transcription and maturation (Eißmann et al., 2012). It plays a crucial role in regulating gene expression in neurons, encompassing nuclear and synaptic functions, as well as synaptogenesis. A study by Masoumi et al. (Masoumi et al., 2019) found that during the peak of EAE and in brain tissue sections of MS patients, MALAT1 expression was downregulated in the central nervous system. A decrease in MALAT1 expression in CD4^+^ T cells promotes the differentiation of these cells into pathogenic Th1 and Th17 phenotypes, while inhibiting Treg differentiation, thereby enhancing the proliferation capability of T cells. These effects indicate that MALAT1 is involved in inducing anti-inflammatory responses. Shaker et al. (Shaker et al., 2019) evaluated the levels of MALAT1 in the serum of 45 Multiple Sclerosis (MS) patients. Compared to the control group, there was a significant increase in MALAT1 levels in the experimental group. The inconsistency in the expression results of MALAT1 across these two studies points to a need for further research in the future to understand these disparities.

LncRNAs have been identified as potential promoters of the progression of MS. For instance, research in the clinical setting has revealed a link between microglia activation and the exacerbation of MS severity (Rothhammer et al., 2018). Microglia play a critical role in demyelination induced by cuprizone through the production of inflammatory cytokines such as TNF-α and interferon-γ (Klein et al., 2018). During this process, the expression of HOTAIR is elevated, which facilitates the transformation of microglia into an inflammatory M1-like phenotype and the secretion of inflammatory mediators, governed by the regulation of miR-136-5p expression (Duan et al., 2018). Duan et al. established that sulfasalazine can reduce the polarization of microglia into the M1 state and curtail the release of inflammatory agents by suppressing HOTAIR expression, thereby aiding in the repair of myelin (Duan et al., 2018). These findings indicate that targeting HOTAIR expression could be a viable strategy in MS treatment protocols (Wang et al., 2022).

Our study does have certain limitations. Firstly, the meta-analysis we conducted included studies primarily from Caucasian populations in Egypt and Iran, lacking data from other ethnic groups like Asians and Africans, which may reduce the representativeness of our findings. Secondly, our research inevitably exhibits significant heterogeneity. Although we identified some major sources of this heterogeneity, the specific sources of heterogeneity in the meta-analysis of lncRNAs for differentiating between relapsing and remitting MS patients remain unclear. Finally, our literature search was confined to English and Chinese language sources, potentially overlooking valuable studies published in other languages, which could introduce some bias into our results.

## Conclusion

LlncRNAs show promise as biomarkers for diagnosing MS and distinguishing between Relapsing and Remitting MS Patients. The biological functions of these markers warrant further investigation to elucidate the molecular mechanisms underlying MS. Moreover, additional large-scale, prospective clinical trials are required to confirm the diagnostic potential of lncRNAs in MS.

## References

[B1] Akbari M. Eshghyar F. Gholipour M. Eslami S. Hussen B. M. Taheri M. (2022). Expression analysis of mTOR-associated lncRNAs in multiple sclerosis. Metab. Brain Dis. 37 (6), 2061–2066. 10.1007/s11011-022-01010-8 35622264

[B2] Amiri M. Mokhtari M. J. Bayat M. Safari A. Dianatpuor M. Tabrizi R. (2022). Expression and diagnostic values of MIAT, H19, and NRON long non-coding RNAs in multiple sclerosis patients. Egypt. J. Med. Hum. Genet. 23 (1), 46. 10.1186/s43042-022-00260-6

[B3] Attia M. S. Ewida H. A. Abdel Hafez M. A. El-Maraghy S. A. El-Sawalhi M. M. (2023). Altered lnc-EGFR, SNHG1, and LincRNA-cox2 profiles in patients with relapsing-remitting multiple sclerosis: impact on disease activity and progression. Diagn. (Basel, Switz.) 13 (8), 1448. 10.3390/diagnostics13081448 PMC10137708 37189549

[B4] Baragilly M. Willis B. H. (2022). On estimating a constrained bivariate random effects model for meta-analysis of test accuracy studies. Stat. Methods Med. Res. 31 (2), 287–299. 10.1177/09622802211065157 34994667 PMC8829734

[B5] Bártulos Iglesias M. Marzo Sola M. E. Estrella Ruiz L. A. Bravo Anguiano Y. (2015). Epidemiological study of multiple sclerosis in La Rioja. Neurologia 30 (9), 552–560. 10.1016/j.nrl.2014.04.016 24975346

[B6] Caraguel C. G. Vanderstichel R. (2013). The two-step Fagan's nomogram: *ad hoc* interpretation of a diagnostic test result without calculation. Evidence-based Med. 18 (4), 125–128. 10.1136/eb-2013-101243 23468201

[B7] Chen L. Guo X. Li Z. He Y. (2019). Relationship between long non-coding RNAs and Alzheimer's disease: a systematic review. Pathol. Res. Pract. 215 (1), 12–20. 10.1016/j.prp.2018.11.012 30470438

[B8] Dahariya S. Paddibhatla I. Kumar S. Raghuwanshi S. Pallepati A. Gutti R. K. (2019). Long non-coding RNA: classification, biogenesis and functions in blood cells. Mol. Immunol. 112, 11282–11292. 10.1016/j.molimm.2019.04.011 31079005

[B9] Dal S. R. Elphick T. L. Fuller K. (2023). Epidemiological study of multiple sclerosis in the Illawarra region. Intern Med. J. 53 (6), 1010–1017. 10.1111/imj.15704 35112760

[B10] Duan C. Liu Y. Li Y. Chen H. Liu X. Chen X. (2018). Sulfasalazine alters microglia phenotype by competing endogenous RNA effect of miR-136-5p and long non-coding RNA HOTAIR in cuprizone-induced demyelination. Biochem. Pharmacol. 155, 155110–155123. 10.1016/j.bcp.2018.06.028 29944870

[B11] Eißmann M. Gutschner T. Hämmerle M. Günther S. Caudron-Herger M. Groß M. (2012). Loss of the abundant nuclear non-coding RNA MALAT1 is compatible with life and development. RNA Biol. 9 (8), 1076–1087. 10.4161/rna.21089 22858678 PMC3551862

[B12] Franco R. Fernández-Suárez D. (2015). Alternatively activated microglia and macrophages in the central nervous system. Prog. Neurobiol. 131, 13165–13186. 10.1016/j.pneurobio.2015.05.003 26067058

[B13] Ghafouri-Fard S. Gholipour M. Eslami S. Hussen B. M. Taheri M. Samadian M. (2023). Abnormal expression of MAPK14-related lncRNAs in the peripheral blood of patients with multiple sclerosis. Noncoding RNA Res. 8 (3), 335–339. 10.1016/j.ncrna.2023.03.006 37091283 PMC10114144

[B14] Ghaiad H. R. Elmazny A. N. Nooh M. M. El-Sawalhi M. M. Shaheen A. A. (2020). Long noncoding RNAs APOA1-AS, IFNG-AS1, RMRP and their related biomolecules in Egyptian patients with relapsing-remitting multiple sclerosis: relation to disease activity and patient disability. J. Adv. Res. 21, 21141–21150. 10.1016/j.jare.2019.10.012 PMC7015469 32071782

[B15] Gharesouran J. Taheri M. Sayad A. Ghafouri-Fard S. Mazdeh M. Omrani M. D. (2019a). A novel regulatory function of long non-coding RNAs at different levels of gene expression in multiple sclerosis. J. Mol. Neurosci. 67 (3), 434–440. 10.1007/s12031-018-1248-2 30610590

[B16] Gharesouran J. Taheri M. Sayad A. Mazdeh M. Omrani M. D. (2019b). Integrative analysis of OIP5-AS1/HUR1 to discover new potential biomarkers and therapeutic targets in multiple sclerosis. J. Cell. Physiol. 234 (10), 17351–17360. 10.1002/jcp.28355 30815864

[B17] Ghoveud E. Teimuri S. Vatandoost J. Hosseini A. Ghaedi K. Etemadifar M. (2020). Potential biomarker and therapeutic LncRNAs in multiple sclerosis through targeting memory B cells. Neuromolecular Med. 22 (1), 111–120. 10.1007/s12017-019-08570-6 31576494

[B18] Gupta M. Martens K. Metz L. M. de Koning A. J. Pfeffer G. (2019). Long noncoding RNAs associated with phenotypic severity in multiple sclerosis. Mult. Scler. Relat. Disord. 36101407, 101407. 10.1016/j.msard.2019.101407 31563073

[B19] Haridy S. Shahin N. N. Shabayek M. I. Selim M. M. Abdelhafez M. A. Motawi T. K. (2023). Diagnostic and prognostic value of the RUNXOR/RUNX1 axis in multiple sclerosis. Neurobiol. Dis. 178106032, 106032. 10.1016/j.nbd.2023.106032 36754216

[B20] Hauser R. M. Henshall D. C. Lubin F. D. (2018). The epigenetics of epilepsy and its progression. Neuroscientist 24 (2), 186–200. 10.1177/1073858417705840 28468530

[B21] Howard J. Trevick S. Younger D. S. (2016). Epidemiology of multiple sclerosis. Neurol. Clin. 34 (4), 919–939. 10.1016/j.ncl.2016.06.016 27720001

[B22] Kamal A. Swellam M. M Shalaby N. Darwish M. K. M El-Nahrery E. (2023). Long non-coding RNAs BACE1-AS and BC200 in multiple sclerosis and their relation to cognitive function: a gene expression analysis. Brain Res. 1814, 148424. 10.1016/j.brainres.2023.148424 37245645

[B23] Klein B. Mrowetz H. Barker C. M. Lange S. Rivera F. J. Aigner L. (2018). Age influences microglial activation after cuprizone-induced demyelination. Front. Aging Neurosci. 10, 278. 10.3389/fnagi.2018.00278 30297998 PMC6160739

[B24] Koch-Henriksen N. Magyari M. (2021). Apparent changes in the epidemiology and severity of multiple sclerosis. Nat. Rev. Neurol. 17 (11), 676–688. 10.1038/s41582-021-00556-y 34584250

[B25] Kortam M. A. Elfar N. Shaker O. G. El-Boghdady N. A. Abd-Elmawla M. A. (2023). MAGI2-AS3 and miR-374b-5p as putative regulators of multiple sclerosis via modulating the PTEN/AKT/IRF-3/IFN-β Axis: new clinical insights. ACS Chem. Neurosci. 14 (6), 1107–1118. 10.1021/acschemneuro.2c00653 36878000

[B26] Labib D. A. Ashmawy I. Elmazny A. Helmy H. Ismail R. S. (2022). Toll-like receptors 2 and 4 expression on peripheral blood lymphocytes and neutrophils of Egyptian multiple sclerosis patients. Int. J. Neurosci. 132 (4), 323–327. 10.1080/00207454.2020.1812601 32842834

[B27] Li J. Zhang Y. Xu Q. Zhang Y. Bei S. Ding Y. (2022). Diagnostic value of circulating lncRNAs for gastric cancer: a systematic review and meta-analysis. Front. Oncol. 12, 1058028. 10.3389/fonc.2022.1058028 36561519 PMC9763557

[B28] Liao Y. Wang R. Wen F. (2022). Diagnostic and prognostic value of long noncoding RNAs in sepsis: a systematic review and meta-analysis. Expert Rev. Mol. diagn. 22 (8), 821–831. 10.1080/14737159.2022.2125801 36106527

[B29] Masoumi F. Ghorbani S. Talebi F. Branton W. G. Rajaei S. Power C. (2019). Malat1 long noncoding RNA regulates inflammation and leukocyte differentiation in experimental autoimmune encephalomyelitis. J. Neuroimmunol. 328, 50–59. 10.1016/j.jneuroim.2018.11.013 30583215

[B30] Miron V. E. Boyd A. Zhao J. W. Yuen T. J. Ruckh J. M. Shadrach J. L. (2013). M2 microglia and macrophages drive oligodendrocyte differentiation during CNS remyelination. Nat. Neurosci. 16 (9), 1211–1218. 10.1038/nn.3469 23872599 PMC3977045

[B31] Negeri Z. F. Beyene J. (2020). Robust bivariate random-effects model for accommodating outlying and influential studies in meta-analysis of diagnostic test accuracy studies. Stat. Methods Med. Res. 29 (11), 3308–3325. 10.1177/0962280220925840 32469266

[B32] Nociti V. Santoro M. (2021). What do we know about the role of lncRNAs in multiple sclerosis. Neural Regen. Res. 16 (9), 1715–1722. 10.4103/1673-5374.306061 33510060 PMC8328773

[B33] Nowak A. Wicik Z. Wolska M. Shahzadi A. Szwed P. Jarosz-Popek J. (2022). The role of non-coding RNAs in neuroinflammatory process in multiple sclerosis. Mol. Neurobiol. 59 (8), 4651–4668. 10.1007/s12035-022-02854-y 35589919

[B34] Rothhammer V. Borucki D. M. Tjon E. C. Takenaka M. C. Chao C. C. Ardura-Fabregat A. (2018). Microglial control of astrocytes in response to microbial metabolites. Nature 557 (7707), 724–728. 10.1038/s41586-018-0119-x 29769726 PMC6422159

[B35] Safa A. Arsang-Jang S. Taheri M. Omrani M. D. Ghafouri-Fard S. (2021). Dysregulation of NF-κB-Associated lncRNAs in multiple sclerosis patients. J. Mol. Neurosci. 71 (1), 80–88. 10.1007/s12031-020-01628-2 32613554

[B36] Sayad A. Taheri M. Arsang-Jang S. Glassy M. C. Ghafouri-Fard S. (2019). Hepatocellular carcinoma up-regulated long non-coding RNA: a putative marker in multiple sclerosis. Metab. Brain Dis. 34 (4), 1201–1205. 10.1007/s11011-019-00418-z 31049796

[B37] Senousy M. A. Shaker O. G. Sayed N. H. Fathy N. Kortam M. A. (2020). LncRNA GAS5 and miR-137 polymorphisms and expression are associated with multiple sclerosis risk: mechanistic insights and potential clinical impact. ACS Chem. Neurosci. 11 (11), 1651–1660. 10.1021/acschemneuro.0c00150 32348112

[B38] Shaker O. G. Mahmoud R. H. Abdelaleem O. O. Ibrahem E. G. Mohamed A. A. Zaki O. M. (2019). LncRNAs, MALAT1 and lnc-DC as potential biomarkers for multiple sclerosis diagnosis. Biosci. Rep. 39 (1). 10.1042/BSR20181335 PMC6331681 30514825

[B39] Soltanmoradi S. Tavakolpour V. Moghadasi A. N. Kouhkan F. (2021). Expression analysis of NF-κB-associated long noncoding RNAs in peripheral blood mononuclear cells from relapsing-remitting multiple sclerosis patients. J. Neuroimmunol. 356, 577602. 10.1016/j.jneuroim.2021.577602 33979709

[B40] Sun D. Yu Z. Fang X. Liu M. Pu Y. Shao Q. (2017). LncRNA GAS5 inhibits microglial M2 polarization and exacerbates demyelination. EMBO Rep. 18 (10), 1801–1816. 10.15252/embr.201643668 28808113 PMC5623836

[B41] Thompson A. J. Banwell B. L. Barkhof F. Carroll W. M. Coetzee T. Comi G. (2018). Diagnosis of multiple sclerosis: 2017 revisions of the McDonald criteria. Lancet Neurol. 17 (2), 162–173. 10.1016/S1474-4422(17)30470-2 29275977

[B42] Tian D. C. Zhang C. Yuan M. Yang X. Gu H. Li Z. (2020). Incidence of multiple sclerosis in China: a nationwide hospital-based study. Lancet Reg. Health West Pac 1, 1100010. 10.1016/j.lanwpc.2020.100010 PMC8315658 34327341

[B43] Wang J. Zhao J. Hu P. Gao L. Tian S. He Z. (2022). Long non-coding RNA HOTAIR in central nervous system disorders: new insights in pathogenesis, diagnosis, and therapeutic potential. Front. Mol. Neurosci. 15949095, 949095. 10.3389/fnmol.2022.949095 PMC9259972 35813070

[B44] Wang P. Xue Y. Han Y. Lin L. Wu C. Xu S. (2014). The STAT3-binding long noncoding RNA lnc-DC controls human dendritic cell differentiation. Science 344 (6181), 310–313. 10.1126/science.1251456 24744378

[B45] Whiting P. F. Rutjes A. W. Westwood M. E. Mallett S. Deeks J. J. Reitsma J. B. (2011). QUADAS-2: a revised tool for the quality assessment of diagnostic accuracy studies. Ann. Intern. Med. 155 (8), 529–536. 10.7326/0003-4819-155-8-201110180-00009 22007046

[B46] Yu Z. Sun D. Feng J. Tan W. Fang X. Zhao M. (2015). MSX3 switches microglia polarization and protects from inflammation-induced demyelination. J. Neurosci. official J. Soc. 35 (16), 6350–6365. 10.1523/JNEUROSCI.2468-14.2015 PMC6605213 25904788

[B47] Zailaie S. A. Siddiqui J. J. Al Saadi R. M. Anbari D. M. S Alomari A. Cupler E. J. (2022). Serum based miRNA as a diagnostic biomarker for multiple sclerosis: a systematic review and meta-analysis. Immunol. Invest. 51 (4), 947–962. 10.1080/08820139.2021.1887888 33660581

[B48] Zamora J. Abraira V. Muriel A. Khan K. Coomarasamy A. (2006). Meta-DiSc: a software for meta-analysis of test accuracy data. BMC Med. Res. Methodol. 631, 31. 10.1186/1471-2288-6-31 PMC1552081 16836745

[B49] Zhao J. Wang Q. Zhu R. Yang J. (2022). Circulating non-coding RNAs as potential biomarkers for ischemic stroke: a systematic review. J. Mol. Neurosci. 72 (8), 1572–1585. 10.1007/s12031-022-01991-2 35380333

